# Characteristic changes to pulsatile and steady‐state load according to pulmonary hypertension classification

**DOI:** 10.14814/phy2.15662

**Published:** 2023-04-11

**Authors:** Sara Hungerford, Katherine Kearney, Ning Song, Eugene Kotlyar, Nicole K. Bart, Edmund Lau, Andrew Jabbour, Christopher Simon Hayward, David William Marshall Muller, Audrey Adji

**Affiliations:** ^1^ Faculty of Health and Medicine The University of New South Wales Sydney New South Wales Australia; ^2^ Department of Cardiology St Vincent's Hospital Darlinghurst New South Wales Australia; ^3^ Department of Cardiology Royal North Shore Hospital Sydney New South Wales Australia; ^4^ Victor Chang Cardiac Research Institute Sydney New South Wales Australia; ^5^ Department of Respiratory Medicine Royal Prince Alfred Hospital Camperdown New South Wales Australia; ^6^ FMHHS Macquarie University Sydney New South Wales Australia

**Keywords:** cardiac magnetic resonance, pulmonary arterial impedance, pulmonary hypertension, right heart catheterization, systemic impedance

## Abstract

It is of increasing importance to understand and predict changes to the systemic and pulmonary circulations in pulmonary hypertension (PH). To do so, it is necessary to describe the circulation in complete quantitative terms. Characteristic impedance (Zc) expresses opposition of the circulation to pulsatile blood flow. Evaluation of systemic and pulmonary Zc relationships according to PH classification has not previously been described. Prospective study of 40 clinically indicated patients referred for CMR and RHC (56 ± 18 years; 70% females, eight mPAP ≤ 25 mmHg, 16 pre‐capillary [Pre‐cPH], eight combined pre‐ and post‐capillary [Cpc‐PH] and eight isolated left‐heart disease [Ipc‐PH]). CMR provided assessment of ascending aortic (Ao) and pulmonary arterial (PA) flow, and RHC, central Ao and PA pressure. Systemic and pulmonary Zc were expressed as the relationship of pressure to flow in the frequency domain. Baseline demographic characteristics were well‐matched across PH subclasses. In those with a mPAP ≤25mHg, systemic Zc and SVR were >2 times higher than pulmonary Zc and PVR. Only Pre‐cPH was associated with inverse pulsatile (systemic Zc 58 [45–69] vs pulmonary Zc 70 [58–85]), but not steady‐state (SVR 1101 [986–1752] vs. PVR 483 [409–557]) relationships. Patients with CpcPH and IpcPH had concordant pulsatile and steady‐state relationships (Graphical Abstract). Measurement of, and the relationship between, systemic and pulmonary Zc in patients according to PH sub‐classification has not previously been described. Systemic Zc was routinely higher than pulmonary Zc, except in patients with newly diagnosed Pre‐cPH, where inverse pulsatile but not steady‐state relationships were observed.

## Graphical abstract

Systemic and pulmonary impedance relationships according to pulmonary hypertension classification.

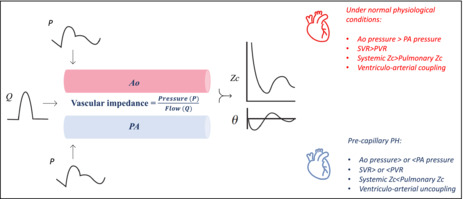

## 1 INTRODUCTION

Over the past decade, there has been renewed interest in uncoupling ventriculo‐arterial interactions due to advances in therapeutics and device technologies which impact upon the properties of arterial circulation. To do so, however, requires describing the arterial circulation in complete quantitative terms—including both steady‐state and pulsatile components. Whereas systemic vascular resistance (SVR) and pulmonary vascular resistance (PVR) are measures of steady‐state load, systemic and pulmonary impedance (Z) express the relationship between pulsatile pressure and flow (Murgo et al., 1980; Murgo & Westerhof, 1984; O'Rourke & Taylor, 1967). Impedance measurement not only provides information about the pulsatile pressure–flow relationships of the arterial circulation but also yields information regarding the physical characteristics of that particular vascular bed and beyond (Morpurgo et al., 1995).

Like the systemic circulation, the pulmonary circulation receives the same blood flow from the heart with the same periodicity. From a hemodynamic point of view, unlike the systemic circulation, however, the pulmonary vascular system is a low‐pressure, low‐resistance, and high‐compliance system with pulsatile energy losses 2.5‐fold greater than that of the systemic circulation (Milnor et al., 1966; Murgo & Westerhof, 1984; O'Rourke, 1982). The relative differences in vascular load between the systemic and pulmonary circulations has created somewhat of a conundrum for clinicians managing patients with pulmonary hypertension (PH) to date.

Until recently, limitations in obtaining simultaneous pulsatile pressure and flow signals in the ascending aorta (Ao) and pulmonary artery (PA) of humans meant that Z estimation was seldom performed outside of the research setting. With advances in noninvasive imaging techniques, the prognostic value of systemic characteristic impedance (Zc) as pulsatile pressure–flow estimation has now been described in healthy human subjects and a variety of cardiovascular disease states using simultaneous applanation tonometry (AT), echocardiographic and/or cardiac magnetic resonance (CMR) techniques (Cramariuc et al., 2009; Hachicha et al., 2007; Hachicha et al., 2009; Hungerford et al., 2021; Jones et al., 2021; McEniery et al., 2008). To date, no reliable method to measure PA pressure noninvasively exists, however, PA flow velocity is readily and accurately obtained by CMR quantitative analysis.

It has previously been shown that PA stiffness may increase early during PH, well before overt elevation of PA pressure occurs (Sanz et al., 2009). More recently, *Hungerford* et al. reported elevated pulmonary Zc to be independent of elevated mean PA pressure and PVR in 70 patients with PH and more strongly predictive of maladaptive RV remodeling than steady‐state indices alone (Hungerford Sara et al., 2023). Deleterious effects of PH on the systemic vascular are also increasingly recognized (Nickel et al., 2020). In this context, there is an urgent and presently unmet need to better predict the response of the RV to adapt to changes in hemodynamic loading conditions—whether it be due to pulmonary vasodilator therapies or transcatheter interventions of the tricuspid valve. Routine, accessible methods to estimate pulmonary Zc are an important key to unlocking this conundrum.

The aim of this study was to measure and compare for the first time, systemic and pulmonary pulsatile (i.e., Zc) and steady‐state (i.e., SVR and PVR) relationships in a cohort of clinically indicated patients undergoing routine right heart catheterization (RHC) and CMR assessment. We hypothesized that the PA impedance spectrum would be qualitatively similar to that of the systemic circulation, albeit with a smaller ratio of peripheral resistance and characteristic impedance, except in those patients with precapillary pulmonary hypertension (Pre‐cPH).

## 2 METHODS

### 2.1 Study population

All new patients with dyspnea and suspected PH referred for RHC over a 12‐month period, with a concurrent clinical indication for CMR, were screened for participation in this study. Forty patients were subsequently enrolled. Indications for CMR in addition to RHC included RV assessment in known or suspected PH, pretransplantation assessment, evaluation of cardiac anatomy and physiology in patients with underlying structural heart disease, and/or advanced heart failure assessment. The most common reasons for exclusion included: unable to complete both RHC and CMR protocol, concurrent pulmonary vasodilator or inotrope therapy (included patients were therefore newly diagnosed and/or treatment naive), evening administration of any medication with a 24‐h systemic vasodilatory effect, significant intracardiac shunt (Qp:Qs > 1.5), or significant (>2+) valvular pathology including tricuspid regurgitation. Morning prescription of any medication with a systemic vasodilatory effect was withheld while the patient was fasted until completion of the RHC/CMR protocol. Each patient underwent same day RHC, followed directly thereafter by CMR.

Precapillary PH (Pre‐cPH) was defined as a mean PA pressure (mPAP) > 20 mmHg with a PVR > 240 dynes s cm^−5^ and mean postcapillary wedge pressure (mPAWP) ≤ 15 mmHg. Isolated postcapillary PH (Ipc‐PH) of left‐sided heart disease was defined as a mPAP > 20 mmHg with a PVR < 240 dynes s cm^−5^ and mPAWP of >15 mmHg. Combined pre‐ and postcapillary PH (Cpc‐PH) was defined as a mPAP > 20 mmHg and PVR > 240 dynes s cm^−5^ and mPAWP > 15 mmHg (Simonneau et al., 2019). Remaining patients not meeting the aforementioned PH criteria were assigned to a mPAP ≤ 25 mmHg group (all had a PVR < 240 dynes s cm^−5^ and mPAWP ≤ 15 mmHg). Local hospital Human Research Ethics committee approved the study. Research was performed in accordance with local hospital guidelines and the Declaration of Helsinki. Informed consent was obtained from all participants prior to enrolment.

### 2.2 Cardiac magnetic resonance image acquisition

CMR studies were performed in a 3T magnet with dedicated phased‐array cardiac coil during successive end‐expiratory breath‐holds (Siemens Magnetom, Erlangen, Germany). Steady‐state free precession (SSFP) images covering the entire LV and RV volumes were first acquired using a standard protocol. In addition, SSFP images were acquired during breath‐hold perpendicular to the ascending aorta and PA. Average scan parameters were repetition time (TR)/echo time (TE) of 3.8/1.6 ms, flip angle 45°, views per segment 8, slice thickness 8 mm, pixel spacing 0.76 mm, acquisition matrix 224 × 192, and temporal resolution 10 ms. Ascending aorta and PA flow velocity data were obtained during breath‐hold at the level of, or prior to, the bifurcation of the main pulmonary artery (MPA) using through plane phase‐contrast quantitative flow (PC Qflow) imaging. Average scan parameters were TR/TE of 4.3–4.6/2.1–2.7 ms, flip angle 15°, slice thickness 8 mm, pixel size 1.25–2.05 mm, acquisition matrix of 256 × 208, views per segment 2, and effective temporal resolution 17 ms. Encoding velocity was adjusted according to known aortic valve gradient or pulmonary artery systolic pressure (sPAP) to minimize aliasing artifact. All CMR analyses were performed using CVI 42 (Circle Cardiovascular Imaging, Calgary, Canada).

### 2.3 Arterial tonometric pressure acquisition

Blood pressure was measured with brachial cuff sphygmomanometer just prior to arterial tonometry (AT) pressure data acquisition. Arterial tonometry was performed using a Millar SPT‐301 high fidelity arterial tonometer (Millar Instruments, Houston, Texas) in the radial artery directly prior to or after CMR scan. Using the SphygmoCor 8.1 system (AtCor Medical, Sydney, Australia), radial AT was calibrated to brachial sphygmomanometer cuff systolic pressures (SP) and diastolic pressures (DP), averaged over 10 cardiac cycles, and then converted to aortic pressure waveforms using a validated transfer function described previously (Adji et al., 2016; Namasivayam et al., 2020).

### 2.4 Right heart catheterization

RHC was performed whilst subjects were fully awake, fasted in the supine position using a standard 7.5 Fr triple lumen Swan‐Ganz catheter (Edwards Lifesciences, Irvine, CA) via the right internal jugular vein. The fluid‐filled catheters and manometer system were routinely calibrated pre‐measurement to ensure reliability. RHC was performed by a single procedural operating team (KK/EK) with more than 15 years of experience. RHC‐derived measurements included right atrial pressure (RAP), sPAP, diastolic PAP (dPAP), mean PAP (mPAP), cardiac output (CO), and pulmonary artery wedge pressure (PAWP). CO measurement was performed using the thermodilution method, expressed as the average of 3–5 consecutive measurements (excluding values with >20% variation). PAWP measurement was performed at end‐expiration, expressed as the average of 3–5 consecutive measurements (mPAWP) (excluding values with >20% variation). Systemic vascular resistance was calculated as: SVR = (mAP [mmHg] − RAP [mmHg]) − CO (L/min) × 80, where *mAP* represents mean arterial pressure, RAP represents right atrial pressure, and *CO* represents cardiac output. Pulmonary vascular resistance (PVR) (expressed as dynes s cm^−5^) was calculated as: PVR = (mPAP [mmHg] − PAWP [mmHg]) − CO (L/min) × 80, where *mPAP* represents mean pulmonary arterial pressure, *PAWP* represents pulmonary artery wedge pressure, and *CO* represents cardiac output obtained by the thermodilution method. The PA pressure waveform was routinely obtained in the main PA 0.5–1 cm distal to the pulmonic valve as confirmed by echocardiography and fluoroscopic assessment. These waveforms were acquired using S5 Collect software with a sampling frequency of 50 Hz. Consecutive signals of PA pressure during 15–20 s without large variation were selected. Pressure signals were divided into each beat according to the R wave of ECG, if available, or the maximum value of the first derivative of PA or RV pressure, followed by ensemble average by 10–15 pressure beats as described previously (Fukumitsu et al., 2020).

### 2.5 Hemodynamic data analysis

Impedance was calculated using frequency domain analysis of the pressure and flow velocity waveforms (Adji et al., 2016; Namasivayam et al., 2020) using Matlab v18 software. Aortic pressure was obtained from AT, while PA pressure was obtained from RHC. Aortic and PA flow velocity waveform data were obtained using CMR PC Q‐flow sequences. Systemic and pulmonary Zc (expressed as dynes s cm^−5^) were then estimated by deconstructing aortic and PA pressure and flow waveforms into their component harmonics for frequencies up to 8–10 Hz, and its modulus averaged between 2 and 10 Hz (Chen et al., 1997; Milnor et al., 1969). Details of this analysis have been described previously (Hungerford et al., 2020; Namasivayam et al., 2020).

### 2.6 Statistical analysis

Data analysis was performed with SPSS‐26 (IBM Corporation, Armonk, New York). Normally distributed variables are presented as means ± SD; nonnormally distributed variables are presented as median (interquartile range [IQR]), unless otherwise specified. Normality was tested by assessing the mean, median, and standard deviation, and a quantile–quantile plot. Student's *t* test or Mann–Whitney *U* test was used to compare variables between patients where appropriate. ANOVA single‐factor analysis was used to compare differences between groups. A Pearson's correlation coefficient calculation was performed to identify any correlation with Zc and other variables. *p* < 0.05 were considered statistically significant.

## 3 RESULTS

The mean age of the study population was 56 ± 18 years, 28 (70%) females. Twenty‐four (60%) patients had evidence of Pre‐cPH or Cpc‐PH on RHC, while eight (20%) had left‐sided heart disease and the remaining eight (20%) patients with a mPAP < 25 mmHg, PVR < 240 dynes s cm^−5^, and mPAWP < 5 mmHg and were assigned to a separate group. Of those with PH, 16 (40%) patients had Pre‐cPH, eight (20%) Ipc‐PH, and eight (20%) had Cpc‐PH. One patient assigned to the Ipc‐PH group had a past history of CTEPH treated successfully with pulmonary endarterectomy, with a more proximate diagnosis of heart failure with preserved ejection fraction (HFpEF). Baseline demographic data are reported in Table 1.

**TABLE 1:** Baseline demographic characteristics.

1a. Median (IQR)	mPAP ≤25 mmHg *n* = 8	PrecPH, *n* = 16	CpcPH, *n* = 8	IpcPH, *n* = 8	*p* value
Age, years	49 (38–57)	52 (41–60)	60 (51–68)	60 (54–64)	All pairwise comparisons *p* > 0.05 (N/S)
Female, *n*	8 (100%)	10 (63%)	6 (75%)	4 (100%)	
Height, cm	161 (158–162)	168 (160–179)	167 (166–175)	171 (162–175)	
Weight, kg	78 (74–82)	73 (60–88)	74 (73–105)	86 (68–96)	
BMI	31 (26–33)	26 (24–31)	28 (23–38)	31 (25–30)	
BSA (m^2^)	1.9 (1.8–1.9)	1.9 (1.6–2.0)	1.9 (1.9–2.2)	2.0 (1.7–2.1)	
HR (bpm)	76 (71–84)	78 (75–81)	68 (67–79)	72 (61–86)	
Brachial SBP (mmHg)	118 (109–119)	119 (107–125)	122 (112–151)	132 (113–124)	
Brachial DBP (mmHg)	68 (61–69)	73 (39–51)	70 (44–67)	57 (69–71)	

1b. Underlying disease mechanism	mPAP ≤25 mmHg *n* = 8	PrecPH, *n* = 16	CpcPH, *n* = 8	IpcPH, *n* = 8
CHD‐PAH	0 (0%)	3 (18.75%)	0 (0%)	0 (0%)
CTEPH	2 (25%)	1 (6.25%)	0 (0%)	0 (0%)
DCM	2 (25%)	0 (0%)	3 (37.5%)	3 (37.5%)
HFpEF	1 (12.5%)	0 (0%)	1 (12.5%)	1 (12.5%)
HTx	0 (0%)	0 (0%)	1 (12.5%)	0 (0%)
ILD	0 (0%)	3 (18.75%)	0 (0%)	0 (0%)
PAH	0 (0%)	9 (56.25%)	2 (25%)	2 (25%)
VHD	0 (0%)	0 (0%)	1 (12.5%)	0 (0%)
Other	3 (37.5%)	0 (0%)	0 (0%)	2 (25%)

Abbreviations: BMI, body mass index; bpm, beats per minute; BSA, body surface area; CHD, congenital heart disease with pulmonary arterial hypertension; CpcPH, combined pre‐ and post‐capillary pulmonary hypertension; CTEPH, chronic thromboembolic pulmonary hypertension; DBP, diastolic blood pressure; DCM, dilated cardiomyopathy; HFpEF, heart failure with preserved ejection fraction; HTx, heart transplantation; ILD, interstitial lung disease; IpcPH, isolated postcapillary pulmonary hypertension; IQR, interquartile range; mPAP, mean pulmonary artery pressure; PAH, pulmonary arterial hypertension; PrecPH, precapillary pulmonary hypertension; SBP, systolic blood pressure; SD, standard deviation; VHD, valvular heart disease (unspecified).

### 3.1 Aortic and pulmonary artery pressure

Radial AT was performed noninvasively in all patients and calibrated to brachial cuff pressure to derive central aortic pressure. There was no significant difference in central mean Ao pressure between mPAP ≤ 25 mmHg patients and those with PH (control 85 [82–86]; Pre‐cPH 90 [80–95]; Cpc‐PH 90 [76–107]; Ipc‐PH 86 [82–87]; *p* = 0.79). There was no significant difference in SVR according to PH subclassification (*p* = 0.96). SVR was highest in patients with Cpc‐PH (1640 [1213–1942] dynes s cm^−5^) and lowest in in patients with Ipc‐PH (1112 [857–1517] dynes s cm^−5^; *p* = 0.96) (Table 2).

**TABLE 2:** Haemodynamic characteristics stratified by PH subclassification.

Median (IQR)	mPAP ≤ 25 mmHg *n* = 8	PrecPH, *n* = 16	CpcPH, *n* = 8	IpcPH, *n* = 8	*p* value*
Function					
Cardiac output (L/min)	5.1 (4.6–5.6)	5.2 (3.8–6.6)	4.5 (4.0–4.8)	5.8 (4.2–6.6)	*p* = 0.73
Cardiac index (L/min/m^2^)	2.8 (2.5–3)	2.9 (2.4–3.6)	2.5 (2.0–2.8)	2.8 (2.2–3.4)	*p* = 0.48
LV ejection duration (cm/s)	284 (247–301)	301 (294–318)	298 (280–300)	322 (281–332)	*p* = 0.73
LV ejection fraction (%)	55 (51–62)	63 (58–67)	56 (46–63)	50 (40–59)	*p* = 0.71
RV ejection duration (cm/s)	311 (283–317)	312 (297–324)	304 (279–318)	278 (259–328)	*p* = 0.50
RV ejection fraction (%)	50 (45–54)	42 (36–49)	46 (37–53)	37 (29–45)	*p* = 0.68
Systemic					
Central mean Ao pressure (mmHg)	85 (82–86)	90 (80–95)	90 (76–107)	86 (82–87)	*p* = 0.79
Central systolic Ao pressure (mmHg)	102 (93–111)	104 (97–115)	115 (95–135)	102 (100–106)	*p* = 0.70
Central diastolic Ao pressure (mmHg)	69 (61–72)	75 (65–79)	76 (64–84)	71 (70–71)	*p* = 0.75
Central Ao pulse pressure (mmHg)	30 (23–35)	32 (28–39)	39 (28–51)	32 (31–32)	*p* = 0.81
Peak Ao flow velocity (cm/s)	75 (63–78)	70 (60–75)	77 (64–82)	73 (55–71)	*p* = 0.67
mPAWP (mmHg)	11 (10–12)	13 (12–14)	25 (23–26)	19 (18–19)	*p* = 0.09
SVR (dyne s cm^−^^5^)	1490 (1095–1490)	1300 (1178–1690)	1640 (1213–1942)	1112 (857–1517)	*p* = 0.96
Systemic Zc (dyne s cm^−5^)	77 (58–106)	58 (45–69)	89 (69–98)	85 (55–90)	*p* = 0.32
Pulmonary					
Central mean PA pressure (mmHg)	21 (18–21)	42 (39–50)	48 (41–52)	26 (25–32)	*p* < 0.01
Central systolic PA pressure (mmHg)	32 (28–36)	66 (54–82)	69 (62–72)	36 (34–44)	*p* < 0.01
Central diastolic PA pressure(mmHg)	12 (10–13)	30 (28–33)	29 (27–36)	18 (17–22)	*p* = 0.01
Central PA pulse pressure (mmHg)	16 (16–22)	37 (29–51)	39 (34–41)	19 (17–20)	*p* = 0.01
Peak PA flow velocity (cm/s)	84 (64–92)	74 (70–82)	69 (60–78)	88 (70–91)	*p* = 0.68
PVR (dyne s cm^−5^)	137 (110–166)	483 (409–557)	372 (344–410)	122 (92–232)	*p* < 0.01
Pulmonary Zc (dyne s cm^−5^)	33 (32–42)	70 (58–85)	63 (45–82)	40 (23–63)	*p* = 0.38

Abbreviations: Ao: aorta; Ao‐Zc: aortic impedance; dPAP: diastolic pulmonary artery pressure; mPAP: mean pulmonary artery pressure; mPAWP: mean pulmonary artery wedge pressure; NS: nonsignificant; PA‐Zc: pulmonary impedance; PVR: pulmonary vascular resistance; RA: right atrial; sPAP: systolic pulmonary artery pressure; SVR: systemic vascular resistance.

* ANOVA single‐factor analysis between all PH subclasses.

**FIGURE 1:**
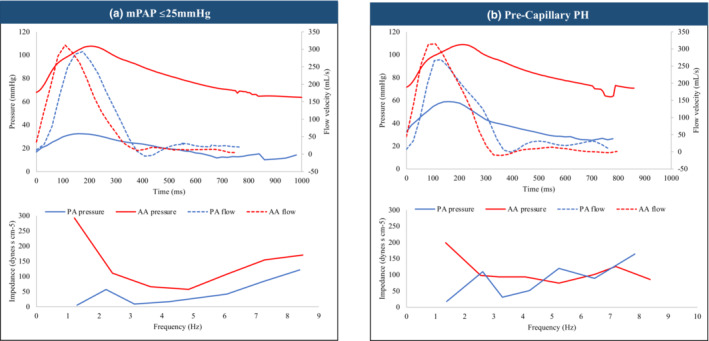
Representative Ao (red) and PA (blue) pressure (solid line) and flow (dotted line) waveform as well as impedance spectra analysis in a study patient (a) with a mPAP ≤ 25 mmHg versus one with Pre‐cPH (b). AA, ascending aorta; mPAP, mean pulmonary artery pressure; PA, pulmonary artery; PH, pulmonary hypertension.

Central PA pressure recorded in the MPA was more than four times lower than central aortic pressure as expected. In patients with a mPAP ≤ 25 mmHg, median mPAP was 21 [18–21] mmHg. Mean PAP was higher in all patients with PH. Mean PAP was highest in patients with Cpc‐PH (48 [41–52] mmHg) and Pre‐cPH (42 [39–50] mmHg) and lowest in patients with left‐sided heart disease from Ipc‐PH (26 [25–32] mmHg; *p* < 0.01). PVR was higher in all patients with PH except for those with Ipc‐PH (mPAP ≤25 mmHg: 137 [110–166] dynes s cm^−5^; Pre‐cPH: 483 [409–557] dynes s cm^−5^; Cpc‐PH: 372 [344–410] dynes s cm^−5^; Ipc‐PH: 122 [92–232] dynes s cm^−5^; *p* < 0.01) (Table 2). Figure 1 shows representative Ao (red) and PA (blue) pressure (solid line) and flow (dotted line) waveforms as well as impedance spectra analysis in a study patient with a mPAP ≤ 25 mmHg (A) versus one with Pre‐cPH (B).

### 3.2 Cardiac magnetic resonance

CMR was performed directly after RHC to maintain a close temporal relationship to minimize any alteration in hemodynamic state. Study patients had no medications or interventions between RHC and CMR. CMR flow velocity data was recorded simultaneously in the Ao and MPA. In the Ao, peak flow velocity was 75 [63–78] cm/s in patients with a mPAP ≤ 25 mmHg; 70 [60–75] cm/s in patients with Pre‐cPH; 77 [64–82] cm/s in patients with Cpc‐PH; and 73 [55–71] cm/s in patients with Ipc‐PH; *p* = 0.67; (Table 2). In the PA, peak flow velocity was 84 [64–92] cm/s in patients with a mPAP ≤ 25 mmHg, versus 74 [70–82] cm/s in patients with Pre‐cPH; 69 [60–78] cm/s in patients with Cpc‐PH; and 88 [70–91] cm/s in patients with Ipc‐PH; (*p* = 0.68; Table 2).

### 3.3 Hemodynamic data analysis

**FIGURE 2:**
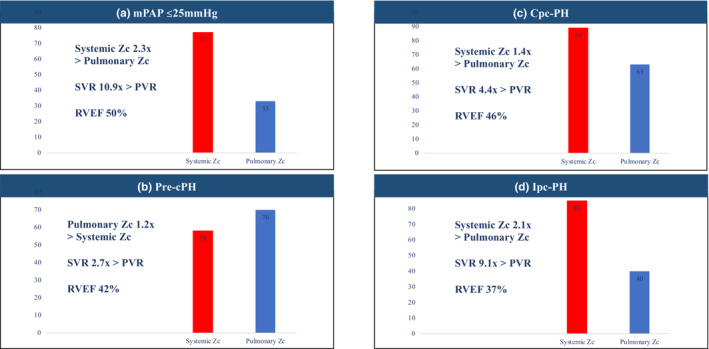
Ensemble comparison of steady‐state (red) and pulsatile (blue) relationships in patients with a mPAP ≤ 25 mmHg (a) and those with PH (b–d). CpcPH, combined pre‐ and post‐capillary pulmonary hypertension; IpcPH, isolated post‐capillary pulmonary hypertension; mPAP, mean pulmonary artery pressure; PA, pulmonary artery; PH, pulmonary hypertension; PrecPH, pre‐capillary pulmonary hypertension; PVR, pulmonary vascular resistance; SVR, systemic vascular resistance; Zc, characteristic impedance.

Pressure–flow analysis permitted quantification of combined systemic and pulmonary Zc to explore interdependent relationships. In patients with a mPAP ≤ 25 mmHg, systemic Zc was 77 [58–106] dynes s cm^−5^ and pulmonary Zc 33 [32–42] dynes s cm^−5^, systemic Zc being 2.3 times higher than pulmonary Zc and SVR 10.9 times higher than PVR. In patients with PH, different hemodynamic relationships were observed. In those with Pre‐cPH, systemic Zc was 58 [45–69] dynes s cm^−5^ and pulmonary Zc was 70 [58–85] dynes s cm^−5^, pulmonary Zc being inversely higher of the two despite SVR being 2.7 times higher than the PVR. This was accompanied by a reduction in RVEF (mPAP ≤ 25 mmHg RVEF 50% vs. Pre‐cPH RVEF 42%; Table 1). No patient with Pre‐cPH had a systemic Zc: pulmonary Zc ratio >2. In those with Cpc‐PH, systemic Zc was the highest at 89 [69–98] dynes s cm^−5^ with pulmonary Zc also elevated at 63 [45–82] dynes s cm^−5^, albeit with a reduced systemic Zc to pulmonary Zc ratio of 1.4. Patients with Ipc‐PH had pseudo normalization of pulsatile and steady‐state relationships. Systemic Zc was 85 [55–90] dynes s cm^−5^ versus 40 [23–63] dynes s cm^−5^ for pulmonary Zc, with systemic Zc 2.1 times greater than pulmonary Zc and SVR 9.1 times greater than PVR (Table 2 and Figure 2).

**FIGURE 3:**
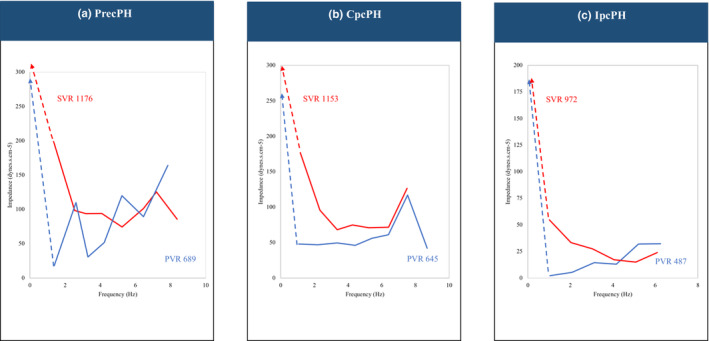
Representative impedance spectra obtained in both vascular beds patients with Pre‐cPH (a), Cpc‐PH (b), and Ipc‐PH (c). AA, ascending aorta; CpcPH, combined pre‐ and post‐capillary pulmonary hypertension; IpcPH, isolated post‐capillary pulmonary hypertension; mPAP, mean pulmonary artery pressure; PA, pulmonary artery; PH, pulmonary hypertension; PrecPH, pre‐capillary pulmonary hypertension.

Figure 3 shows representative impedance spectra obtained in both vascular beds from different patient populations. Although marked similarities in overall shape of the pressure spectra are present, it is apparent that all harmonics of systemic pressure were of a greater magnitude than the corresponding harmonics of PA pressure, except for patients with Pre‐cPH. Except for the mean flow terms, which by necessity and calibration techniques, are set equal to each other, all of the harmonics of the pulmonary flow moduli were generally lower in magnitude than the respective harmonics of the systemic flow moduli except for those with Pre‐cPH. The impedance moduli of the systemic circulation appeared to oscillate more than the impedance moduli of the pulmonary circulation, with the exception of patients with Pre‐cPH. The phase components of the PA input impedance were less negative than the Ao input impedance spectra.

## 4 DISCUSSION

The findings of this study can be summarized as follows: (Murgo et al., 1980) pulsatile and steady‐state values were higher in the systemic than the pulmonary circulation, except for patients with Pre‐cPH; (Murgo & Westerhof, 1984) newly diagnosed Pre‐cPH was uniquely associated with inverse pulsatile, but not steady‐state, relationships of the systemic and pulmonary circulations; (O'Rourke & Taylor, 1967) patients with Ipc‐PH had pseudo normalization of pulsatile and steady‐state relationships, and; (Morpurgo et al., 1995) amplitudes of the harmonics of pressure and flow were generally smaller for the pulmonary circulation, except for patients with Pre‐cPH. These findings are consistent with the lower pressures and more rounded waveforms known to be characteristic of the pulmonary circulation from previous invasive human studies (Laskey et al., 1993; Milnor et al., 1969; Nichols et al., 1977), but this study is the first to demonstrate the characteristic systemic and pulmonary circulatory relationships that exist across different PH populations.

### 4.1 Evolution of methods to determine vascular impedance

In recent years, there has been renewed interest in more accurate quantification of the pulmonary circulation in patients with PH. Use of prostacyclin and its analogs, with inhaled nitric oxide, endothelin antagonists, and phosphodiesterase inhibitors are known to reduce steady‐state load, however, considerably less has been published on the pulsatile pressure and flow phenomena that are thought to occur with these therapeutics. This is important, as unlike the systemic circulation where relative changes in steady‐state and pulsatile load are far smaller (a 25% increase in mean arterial pressure is considered severe), the mean PA pressure can increase fourfold in patients with PH and this is accompanied by arterial dilatation and disturbed flow–velocity profile (Chemla et al., 2002).

In general terms, Z is calculated from the ratio of the pressure difference across the system under study and the flow through the system (Murgo et al., 1980). As outlined above, this descript of the arterial system (found by only taking the ratio of mean pressure difference and mean flow as in the case of SVR or PVR) is limited as it only gives a value of peripheral resistance at steady state. Accurate quantification of the pulmonary circulation requires measurement of Zc by acquiring pulsatile pressure and flow waves and deriving their respective sinusoidal components by Fourier analysis. In the past decade, there have been significant advances in the noninvasive measurement of systemic Zc in healthy human subjects (Adji et al., 2016) and cardiovascular disease states (Hungerford et al., 2020; Namasivayam et al., 2020) using a simultaneous CMR and AT technique (the latter from which central aortic pressure can be derived from a radial tonometer). This approach is not easily translated to the pulmonary circulation, however, as no reliable noninvasive measure of PA pressure exists, although efforts are underway (Ramos et al., 2022). Most existing parameters of PA stiffness (including PA compliance, distensibility, capacitance, elasticity, and stiffness index) still require a combined approach of invasive measurement of intrapulmonary pressures with RHC and noninvasive measurement of change in PA diameter or cross‐sectional area within the cardiac cycle on CMR (Freed et al., 2016). Completely noninvasive measures of PA stiffness (including comparison of PA diameter with aortic diameter and pulsatility), have not been demonstrated to be significantly different in patients with low or high PVR (Gupta et al., 2018). To the best of our knowledge, this is the first study to coalesce readily available Ao and PA pressure and flow velocity data to determine systemic and pulmonary Zc using frequency domain analysis in patients according to PH subclassification (see Figure 1). CMR is widely considered to be the noninvasive “gold standard” for estimation of arterial flow (Tadic, 2015), while PA pressure waveforms are routinely collected during routine RHC evaluation as we have demonstrated.

### 4.2 Unique characteristics of the systemic and pulmonary circulations

Relative to the systemic circulation, the normal pulmonary vascular bed is a low‐pressure, low‐resistance, and high‐compliance system capable of accommodating large increases in blood flow with minimal elevations of PA pressure (Verhoeff & Mitchell, 2017). From a hemodynamic point of view, there are a number of key differences between the right and left heart circulations. Firstly, the mPAP, PVR, and pulse pressure are about one sixth that of the systemic circulation (Lankhaar et al., 2006). Secondly, the main PA artery is highly distensible with no progressive increase in stiffness between central and distal sites as seen in the systemic circulation (Attinger, 1963). Thirdly, impedance moduli of the systemic circulation are also known to oscillate more than that of the pulmonary circulation. Finally, phase components of PA input impedance spectra have also been demonstrated to be less negative than the aortic input impedance spectra (Murgo & Westerhof, 1984). In a seminal historical study of “normal” human subjects conducted by *Murgo* et al. over half a century ago, PVR was demonstrated to be approximately 8% lower than SVR, and pulmonary Zc approximately 43% lower than systemic Zc (Murgo & Westerhof, 1984). The hemodynamic values obtained in this study are reassuring in that regard. In patients with a mPAP ≤25 mmHg, systemic Zc was more than twofold higher than pulmonary Zc, although SVR was more than 10‐fold higher than PVR. With the exception of the mean flow terms, which by necessity and calibration techniques are set equal to each other, all of the harmonics of the PA flow moduli were lower in magnitude than the respective systemic harmonics in the mPAP ≤25 mmHg cohort as demonstrated previously (Murgo & Westerhof, 1984) (see Figure 2).

### 4.3 Impedance patterns in healthy individuals and pulmonary hypertension disease

Impedance estimation is highly sensitive to any changes to the human circulation, either within a given subject under certain physiological conditions, as a result of pharmacological interventions, or between groups of subjects under different physiological or pathological states (Laskey et al., 1993). In physiological conditions, the heart is coupled to the arterial circulation by relative matching between contractility and afterload. In patients with a mPAP ≤ 25 mmHg in this study, a median pulmonary Zc of 33 [32–42] is in the same order of magnitude as previous historical invasive studies when adjusted for patient age and baseline mPAP (Milnor et al., 1969; Murgo & Westerhof, 1984).

As PH develops, the vascular bed becomes a high‐pressure, high‐resistance, and low‐compliance system (Chemla et al., 2002). The effect of these hemodynamic changes is to impart additional load on the right ventricle and alter efficiency of ventriculo‐arterial (VA) coupling. To preserve efficient VA coupling, the right ventricle must adapt to the chronic increase in afterload with a compensatory hypertrophy. Eventually, the sustained pressure overload encumbers performance and right ventricular dysfunction and/or dilatation ensue. RV‐PA decoupling predicts poor outcomes in patients referred for PH (Kussmaul et al., 1988; Murgo et al., 1980; Vanderpool et al., 2018; Vonk Noordegraaf et al., 2017). The pathogenicity of this process highlights an urgent and presently unmet need to identify changes to the pulsatile circulation in its earliest stages (even prior to elevation of central pressure or PVR).

In this study, unique pressure–flow relationships were observed in patients with PH. For patients with Pre‐cPH, a pulmonary Zc value of 70 [58–85] dynes s cm^−5^ is comparable with a prior invasive study by *Laskey* et al. (Laskey et al., 1993), however, we also found that pulmonary Zc was independently 1.2 times higher than systemic Zc (despite SVR being 2.7 times higher than PVR), with marked oscillations of impedance moduli. Interestingly, systemic Zc was nonsignificantly lower in patients with Pre‐cPH compared to all other cohorts. There is growing evidence to suggest that patents with Pre‐cPH exhibit systemic vascular dysfunction (including impaired brachial artery flow‐medial dilatation, abnormal cerebral blood flow, skeletal myopathy, and intrinsic kidney disease) due to a milieu of metabolic and endocrine abnormalities (Nickel et al., 2020). Although the findings of this study are inconclusive, further comparative analysis of pulmonary and systemic Zc in patients with advanced PH is warranted. On the other hand, in those with Cpc‐PH (with a significant increase in pulmonary venous pressure), systemic and pulmonary Zc were both elevated in this study, but with relative preservation of pulsatile (systemic Zc 1.4 times greater than pulmonary Zc) and steady‐state (SVR 4.4 times greater than PVR) relationships (see Figure 2 and Table 2).

Aside from study of pulmonary Zc patterns in healthy individuals and Pre‐cPH, limited work has been done to evaluate pulmonary Zc in patients with PH secondary to mitral stenosis or left ventricular failure. Patterns of pulmonary Zc in patients with mitral stenosis and/or left ventricular failure have previously demonstrated an increase in the resistive component of modulus, an increase in frequency of the first minimum of the modulus and of phase cross over, as well as an increase in pulmonary Zc (Chen et al., 1990; Kussmaul et al., 1992, 1988; Milnor et al., 1969). These findings were also observed of our cohort with Ipc‐PH. In patients with Ipc‐PH or isolated left‐sided heart disease, systemic Zc was 2.1 times higher than pulmonary Zc resembling pseudo‐normalization of pulsatile and steady‐state relationship, with the main difference being a higher relative systemic and pulmonary Zc values.

### 4.4 Limitations

We have developed a hybrid method by which systemic and pulmonary Zc may be measured during routine CMR and RHC evaluation. Our method combines “gold standard” invasive and noninvasive tools to measure pressure, volume, and flow, which are then combined to provide high‐quality impedance assessment. Nonetheless, some limitations do exist. Six patients in the mPAP ≤ 25 mmHg had a mPAP between 20 and 25 mmHg, meaning we were not able to assign this group as a “healthy” control population. As per inclusion criteria, patients were not on any concurrent pulmonary vasodilator, inotrope therapy, or systemic vasodilatory therapy at time of study and were therefore, in most part, newly diagnosed and/or treatment naive. We were not able to perform RHC simultaneously with CMR due to technical constraints. It is possible that an alteration in hemodynamic loading conditions occurred between RHC and CMR study that may have affected results to an extent, however, every care was taken to ensure stable supine resting conditions for both measurements and no significant difference in HR or BP between RHC and CMR was observed (both *p* > 0.05; Table 1). We did not perform distal Zc or PWV analysis as we might have liked due to procedural constraints. High‐fidelity PA catheters may have provided a higher frequency waveform for more accurate measurement but limit real‐world reproducibility of the technique. Clinical studies are currently underway to validate CMR measurement of PA pressure (Ali et al., 2022). Once a reliable method of PA pressure measurement by CMR is validated, future application of our CMR/AT technique is expected to provide complete noninvasive assessment of systemic and pulmonary Zc.

## 5 CONCLUSIONS

This study has demonstrated for the first time, inverse pulsatile, but not steady‐state, relationships in patients with newly diagnosed Pre‐cPH. The value of routine Zc measurement in better defining patient prognosis, and of predicting clinical outcomes of medical and interventional therapies remains to be determined. What is clear, is that the newfound ease of noninvasive Zc estimation by CMR, is expected to yield further information about systemic/pulmonary relationships, above and beyond conventional resistance indices alone.

## AUTHOR CONTRIBUTIONS

Sara Hungerford, Katherine Kearney, and Ning Song: All aspects of study writing and design. Eugene Kotlyar, Nicole K. Bart, Edmund Lau, Andrew Jabbour, Christopher Simon Hayward, David William Marshall Muller: Study design, supervisor support, and review of article. Audrey Adji: All aspects of study writing and design.

## ETHICS STATEMENT

The study was approved by the local hospital Human Research and Ethics Committee.

## FUNDING INFORMATION

Dr Katherine Kearney and Dr Ning Song are supported by an Australian Government research training program grant. All other authors have reported that they have no funding disclosures relevant to the contents of this article to disclose.

## CONFLICT OF INTEREST STATEMENT

All authors have reported that they have no relationships relevant to the contents of this paper to disclose.
